# Soft Clustering for Enhancing the Diagnosis of Chronic Diseases over Machine Learning Algorithms

**DOI:** 10.1155/2020/4984967

**Published:** 2020-03-09

**Authors:** Theyazn H. H Aldhyani, Ali Saleh Alshebami, Mohammed Y. Alzahrani

**Affiliations:** ^1^Department of Computer Sciences and Information Technology, King Faisal University, Al-Hasa 31982, Saudi Arabia; ^2^Department of Administrative and Financial Sciences, King Faisal University, Al-Hasa 31982, Saudi Arabia; ^3^Department of Computer Sciences and Information Technology, Albaha University, Albaha 65527, Saudi Arabia

## Abstract

Chronic diseases represent a serious threat to public health across the world. It is estimated at about 60% of all deaths worldwide and approximately 43% of the global burden of chronic diseases. Thus, the analysis of the healthcare data has helped health officials, patients, and healthcare communities to perform early detection for those diseases. Extracting the patterns from healthcare data has helped the healthcare communities to obtain complete medical data for the purpose of diagnosis. The objective of the present research work is presented to improve the surveillance detection system for chronic diseases, which is used for the protection of people's lives. For this purpose, the proposed system has been developed to enhance the detection of chronic disease by using machine learning algorithms. The standard data related to chronic diseases have been collected from various worldwide resources. In healthcare data, special chronic diseases include ambiguous objects of the class. Therefore, the presence of ambiguous objects shows the availability of traits involving two or more classes, which reduces the accuracy of the machine learning algorithms. The novelty of the current research work lies in the assumption that demonstrates the noncrisp Rough K-means (RKM) clustering for figuring out the ambiguity in chronic disease dataset to improve the performance of the system. The RKM algorithm has clustered data into two sets, namely, the upper approximation and lower approximation. The objects belonging to the upper approximation are favourable objects, whereas the ones belonging to the lower approximation are excluded and identified as ambiguous. These ambiguous objects have been excluded to improve the machine learning algorithms. The machine learning algorithms, namely, naïve Bayes (NB), support vector machine (SVM), K-nearest neighbors (KNN), and random forest tree, are presented and compared. The chronic disease data are obtained from the machine learning repository and Kaggle to test and evaluate the proposed model. The experimental results demonstrate that the proposed system is successfully employed for the diagnosis of chronic diseases. The proposed model achieved the best results with naive Bayes with RKM for the classification of diabetic disease (80.55%), whereas SVM with RKM for the classification of kidney disease achieved 100% and SVM with RKM for the classification of cancer disease achieved 97.53 with respect to accuracy metric. The performance measures, such as accuracy, sensitivity, specificity, precision, and *F*-score, are employed to evaluate the performance of the proposed system. Furthermore, evaluation and comparison of the proposed system with the existing machine learning algorithms are presented. Finally, the proposed system has enhanced the performance of machine learning algorithms.

## 1. Introduction

Chronic diseases are serious diseases because they pose a serious threat to people's lives and persist over long periods. They can impede the freedom and health of people who have physical disabilities. Thus, they further cause frustration of people who suffer from various health disabilities. The available vaccines and medicine cannot completely prevent chronic diseases because they show no indications in any case. With aging changes, chronic diseases continue to become a more common phenomenon. Hence, there is a need to identify factors causing them and to take the required corrective measures accordingly. Factors such as smoking, physical inactivity, food diet, and insufficient or excessive alcohol consumption could largely contribute to chronic diseases. Previous studies have identified chronic diseases as the seventh cause of death among other causes. In the United States, they resulted in 65.8% of deaths among US males and 67.2% of deaths among US females in 2010 [1]. Heart, cancer, diabetes, asthma, and kidney diseases are identified as chronic diseases. In addition, chronic diseases are measured as noncommunicable diseases; they slowly end the life of people in a long period. Chronic diseases do not transfer from one person to another. In the United States, chronic diseases drive up medicinal service expenses and break up human services reasonably. They possess an essential part of the economy and thwart the health quality of people. This study promotes the classification of chronic disease conditions, namely, cancer, kidney, asthma, and diabetes. The World Health Organization reported in 2002 that mortality, dreariness, and incapacity were credited to the major chronic diseases. Currently, records show that 60% of all deaths and 43% of the global weight of illnesses are attributed to chronic diseases. By 2020, it is expected that the percentage of deaths will reach 73% of total deaths and 60% of the global weight of sicknesses [2]. With the help of machine learning algorithms, predicting chronic diseases has become an easy task. Therefore, it is aimed here to develop a surveillance system to predict and diagnose healthcare data for helping the health communities.

The machine learning algorithms increase the level of individual prediction, and therefore diagnosis will ultimately strengthen anticipation efforts. The availability of well prevention measures will not only enhance or provide good health for persons but also reduce healthcare spending. Machine learning techniques have been used widely in the healthcare domain. They have now become crucial tools for healthcare management. They have also assisted in improving health care by using the prediction measures for epidemic diseases faced by people around the world. The World Health Organization (WHO) has significantly benefited from the employment of machine learning applications that improve the quality of health care.

Machine learning algorithms are considered to be classification, clustering, and prediction for the sake of solving various issues in real-time applications. They provide an assurance of the classification and prediction solutions for stability and reliability in performance. Based on machine learning algorithms, a few researchers have developed successful healthcare systems. Algorithms include statistics, SVM, decision trees, clustering, and optimization algorithms and others. Machine learning applications rely largely on datasets that analyze and discover the patterns that are used to solve specific tasks. The healthcare system has the potential promotion in the health domain to extract and discover the hidden patterns in the database [3]. Thus, the available healthcare data are universally scattered and ambiguous. They may also contain insufficient and insignificant information stored in terms of the constancy in prediction and classification. One of the biggest challenges of healthcare data and its information is the accurate diagnosis of certain significant information. To predict and analyze the chronic diseases such as kidney, diabetic, cancer, and heart diseases, there are several proposed machine learning algorithms that can be used. These algorithms include the decision tree (DT), SVM, ANN, linear regression (LR), KNN, NB, and time series prediction models. Because of the rapid innovation and continuous changes in software engineering, a huge volume of information can be generated. With the development of a healthcare database management system, there will be more opportunities for the enhancement of the healthcare systems. Extracting patterns from these datasets and managing large amounts of dimensionality data have become a major field of machine learning. The machine learning algorithm is considered to be the classification of healthcare datasets to obtain useful knowledge that can help health officials and communities. To apply machine learning algorithms that enhance the performance of the classification process, the preprocessing of the soft clustering algorithm is required.

The remaining parts of the article are organized into sections. Introduction is discussed in Section 1, related studies are given in Section 2, data and methods are shown in Section 3, and results and discussion are shown in Section 4. Lastly, conclusion is presented in Section 5.

## 2. Related Studies

There is a considerable number of research works that have been done in relation to the classification and the prediction of healthcare data. Solanki [4] proposed most of the classifier algorithms on the Weka tool for predicting the prevalent sickle cell disease. The obtained results compared with the classifiers are available on the Weka data mining tool. It is observed that the random tree approach is a better algorithm for classifying sickle cell. Similarly, Joshi et al. [5] used a number of machine learning approaches such as Bayes net, logistic model tree (LMT), multilayer perception, stochastic gradient descent, and sequential minimal optimization techniques. These researchers suggested using LMT algorithms for diagnosing breast cancer because of its high performance and accuracy. Furthermore, David et al. [6] applied the KNN algorithm, Bayesian network, decision tree algorithm, and random tree method, namely, the J48 tree to predict leukemia disease. Accordingly, it was found that the decision tree algorithm had shown better accuracy in the result. In one more study conducted by Vijayarani and Sudha [7], LMT and the sequential minimal optimization multilayer, and perceptron algorithms are employed to predict heart diseases. Furthermore, the study conducted by Sugandhi et al. [8] proposed a random tree algorithm for the classification of heart diseases. The outcome of the research has shown that random tree gives a better performance than other classification algorithms. Consequently, for obtaining results from a random tree classifier, it is found that the random tree classifier is outperformed. The study of Yasodha and Kannan [9] has also reported that the Weka classification algorithm was used for analyzing and predicting the diabetic patient's database. Likewise, Bin Othman and Yau [10] have compared various classification approaches with the Weka data mining tool for predicting breast cancer. Israa [11] has applied NB, decision tree (DT), random forest, and support vector machine techniques to improve the classification of heart diseases. On the other hand, D. Sisodia and Sisodia [12] have proposed three machine learning algorithms; that is, DT, SVM, and naive Bayes (NB) to detect diabetes. Thus, experiments have been done by using standard data from the UCI machine learning repository. It is observed that the NB approach outperforms as compared with other algorithms, which have an accuracy rate of 76.30%. The study of Syed et al. [13] has employed SVM, Bayesian network, and decision tree algorithms to predict the obesity of schoolchildren. Sandeep et al. [14] proposed linear discriminate analysis (LDA), NB, random forest, LR, and quadratic discriminate analysis (QDA) for the analysis and classification of chronic kidney diseases. The study of Sahana and Minavathi [15] has also focused on predicting kidney disease using classification algorithms, namely, ANN and C45. It concentrated on accurate prediction and time factor performance. The ANN and C45 algorithms are used for helping out the medical practitioner to give proper medication and medical treatment. K. Polaraju and Prasad [16] have proposed a multiple regression model to classify chronic heart disease. It is proved that the multiple linear regression model is favourable for predicting heart diseases.

In this research, the training dataset consists of 3000 values with 13 different features. From the experimental results, it is shown that the regression algorithm performs better than other algorithms. Kim et al. [17] have proposed the character-recurrent neural network (Char-RNN) model to predict chronic diseases. They have collected data from the Korean National Health and Nutrition Examination Survey (KNHANES). It is observed that the Char-RNN model obtained higher accuracy than the conventional multilayer perceptron model. Ng et al. [18] have used machine learning algorithms to detect heart failure. Moreover, electronic health record data are used to predict events and the onset of diseases. Zhang et al. [19] have proposed a convolution neural network (CNN) architecture named Group Net to predict chronic diseases. Thus, the experimental analysis is conducted using data from local medical centers. They have noted that CNN has achieved the best accuracy. Kriplani et al. [20] have used deep learning to predict chronic kidney disease. The proposed models are tested by using standard datasets of diseases available on the UCI. The analysis results have appeared better in using cross-validation performance. Liu et al. [21] proposed CNN, LSTM, and hierarchical models to predict chronic diseases. Brisimi et al. [22] have applied four machine learning algorithms, namely, SVM, kernelized, sparse logistic regression, and random forests to predict chronic heart and diabetic diseases. They have gathered standard data from electronic health records (EHRs). Chen et al. [23] used streamline machine learning techniques to predict chronic disease epidemics. Their experiment has proposed prediction models using real-life hospital data gathered from central China in 2013–2015. The convolution neural network is implemented as based on multimodal disease risk prediction (CNN-MDRP) algorithm using structured and unstructured data from hospitals. Patel et al. [24] have developed a system using three classifiers such as KStar, SMO, and J48, Bayes net, and multilayer perception neural network algorithms with the help of Weka software to classify heart diseases. It is observed that the Bayes net has accomplished optimum performance as compared with further classification algorithms, namely, KStar, multilayer perception, and J48 approaches by using the k-fold cross-validation method. The research of Deepika and Seema [25] also designed a system to predict chronic diseases via machine learning algorithms such as naïve Bayes, decision tree, SVM, and ANN. A comparative analysis of the performances of algorithms is presented. It is observed that the support of the vector machine and the naïve Bayes provides the highest accuracy rate when predicting the diabetic disease. Ul Haq et al. [26] have suggested different machine learning algorithms such as naive Bayes, classification tree, KNN, logistic regression, SVM, and ANN to predict heart diseases. Three feature selection methods are applied to improve the classification algorithms. It is concluded that the feature selection method increases the performance of the classifier for predicting heart diseases. Ahmed et al. [27] proposed a fuzzy logic algorithm to classify kidney diseases. Coacci et al. [28] used two classification approaches, namely, logistic regression and ANN. Xun et al. [29] presented ANN and naïve Bayes classifiers for predicting chronic diseases. Some researchers used the UCI machine learning repository for testing proposed models, such as chronic diseases [30, 31], diabetic disease [32, 33], and breast cancer [34]. Using the deep learning algorithm to predict chronic diseases, Kim et al. [17] proposed nature-inspired computing algorithms for the diagnosis of chronic diseases [35] and employed machine learning algorithm to develop E-health for the diagnosis of chronic diseases [36].

In the current research article, traditional machine learning algorithms are employed for predicting chronic diseases. Therefore, the result of the existing classification algorithms is needed to make the healthcare system more reliable. Subsequently, the soft clustering algorithm is applied to increase the accuracy of classification algorithms.

## 3. Materials and Methods

**Figure 1:**
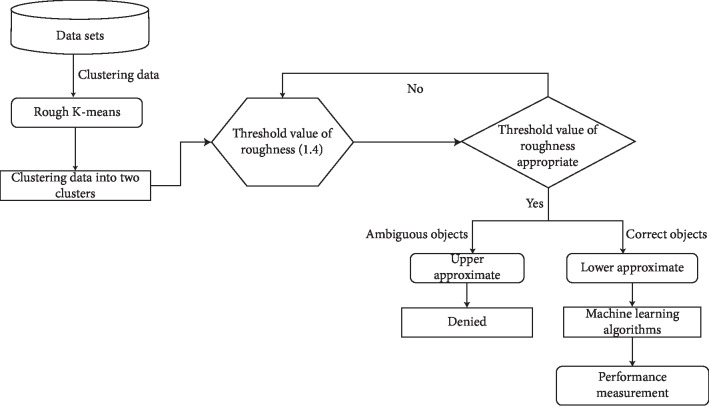
Proposed model.

The proposed model is designed explicitly to classify chronic diseases using machine learning algorithms.  Figure 1 displays the proposed system that combines the existing machine learning algorithms with the rough k-means clustering technique. Noncrisp rough k-means algorithm is demonstrated to handle the ambiguous objects. These ambiguous objects obstruct the performance of machine learning algorithms. The RKM clustering has clustered data into two clusters. Thus, it is used to measure the roughness of the objects. Moreover, the threshold value is also used to maximize the roughness of objects for reducing the ambiguous objects. The threshold value parameter plays a very significant role in making the program of the noncrisp algorithm. It has experimented and found out that the threshold value is 1.4. The RKM algorithm is used to deal with ambiguous objects for improving the classification algorithm. The RKM algorithm has clustered data into lower approximation and upper approximation in which the clustered objects in the lower approximation are considered, but the objects clustered in upper approximation are excluded. The novelty of the proposed model has used rough k-means to handle the ambiguous objects belonging to lower approximation that is processed with the help of machine learning algorithms. The rough k-means clustering is proposed to explicitly determine ambiguous objects. To close, it is investigated that the results of the proposed system have outperformed all the alternative models used for measuring the performance. The detailed description of the proposed system is discussed in the following subsections.

### 3.1. Datasets

The chronic disease datasets have been collected from the different resources as follows:

#### 3.1.1. Diabetic Disease Dataset

The diabetes data collected from the machine learning repository contained nine attributes, eight features, and one class. This dataset has been gathered from an automatic electronic recording device and paper records [37].  Table 1 shows the features of data.

#### 3.1.2. Breast Cancer Disease Dataset

The cancer data collected from the Kaggle contained nine attributes, 30 features, and 1 class. These features are obtained from digitized images of breast cancer [38].  Table 2 shows the features of data.

#### 3.1.3. Kidney Disease Dataset

The collected kidney data from the Kaggle contained 26 attributes, 24 features, and 1 class [38].  Table 3 shows the features of data.

### 3.2. Handling Ambiguity

**Figure 2:**
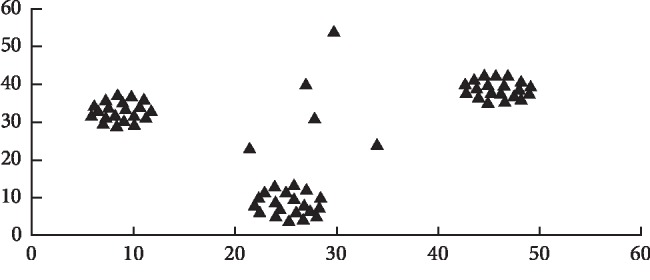
Sample of ambiguous objects.

Machine learning algorithms have succeeded in a number of real-time applications such as image processing recognition, video recognition, marketing prediction, weather forecasting, and network security. The conventional machine learning algorithms are used to identify the objects belonging to exactly one class. In data analysis, it may be possible that an object shows the characteristics of different classes [39]. In that event, an object should belong to more than one class, and as a result, object boundaries should necessarily overlap. The machine learning algorithms categorize an object into one class precisely.  Figure 2 shows the ambiguous data. Such requirement is found to be too restrictive in a number of real-time applications.

In Figure 2, the basic example of ambiguous objects can be noted. It clearly shows the three separate classes. Hence, it is observed that five objects are not classified under any precise class. Henceforth, these five objects decrease the performance of machine learning algorithms. Thus, it is required to determine such ambiguous objects and deal with them before applying machine learning algorithms. For this issue, the present research work applies the RKM technique to recognize ambiguous packets from chronic disease datasets. The detailed description of the RKM algorithm employed for identifying the ambiguous objects is presented in the subsequent subsections.

#### 3.2.1. Rough K-Means Clustering Algorithm

The proposed RKM clustering approach is based on a simple K-means clustering [40–42]. Peters [43] enhanced the algorithm of [40] (original proposal) by calculating rough centroid using ratios of distances as new proposals to differentiate between similar distances. Joshi and Lingras [44] used RKM and ECM clustering algorithms to handle high dimensional data. Aldhyani and Joshi [39] used the rough K-means and ECM clustering algorithms to handle ambiguous objects of intrusion detection. The rough K-means approach is designed to determine the ambiguous objects that belong to the upper boundary of clusters. Cluster the data as lower approximation and upper approximation. The rough K-Means represents each.

  (P1) An object $\overset{⟶}{x}$ can be part of, at most, one lower approximation (lower bound)
  (P2) $\vec{x}$ ∈ $\underline{A}\left({\overset{⟶}{c}}_{i}\right)$ = ⇒$\overset{⟶}{c}$ ∈ $\bar{A}\left({\overset{⟶}{c}}_{i}\right)$
  (P3) An object $\overset{⟶}{x}$ is not part of any lower approximation
 
⇕
 
$\overset{⟶}{x}$ belongs to two or more upper approximations (upper bound)

Overall, ideas of soft clustering are more appropriate to deal with ambiguous objects. When the algorithm is processed, all objects are assigned *w*_lower_ and *w*_upper_. For each object vector, $\overset{⟶}{v}$ let *d* ($\overset{⟶}{v}$, ${\overset{⟶}{c}}_{j}$) be the distance between itself and the centroid of cluster ${\overset{⟶}{c}}_{j}$. Let *d* ($\overset{⟶}{v}$, ${\overset{⟶}{c}}_{i}$) = min 1 ≤ *j* ≤ *k d* ($\overset{⟶}{v}$, ${\overset{⟶}{c}}_{j}$). The ratios *d* ($\overset{⟶}{v}$, ${\overset{⟶}{c}}_{j}$)/*d* ($\overset{⟶}{v}$, ${\overset{⟶}{c}}_{j}$), 1 ≤ *I*, *j* ≤ *k*, are used to determine the membership of $\overset{⟶}{v}$. Let *T* = {*j* : *d* ($\overset{⟶}{v}$, ${\overset{⟶}{c}}_{j}$)/*d* ($\overset{⟶}{v}$, ${\overset{⟶}{c}}_{j}$) ≥ threshold and *i* ≠ *j*}.

1. If *T* = *ϕ*, $\overset{⟶}{v}$ ∈ *A* (${\overset{⟶}{c}}_{j}$) and $\overset{⟶}{v}$ ∈ *A* (${\overset{⟶}{c}}_{j}$), ∀*j* ∈ *T*. Furthermore, $\overset{⟶}{v}$ is not part of any lower approximation? The above criterion guarantees that property (P3) is satisfied.
2. Otherwise, if *T* = *ϕ*, $\overset{⟶}{v}$ ∈ *A* (${\overset{⟶}{c}}_{j}$). In addition, by property (P2), $\overset{⟶}{v}$ ∈ *A* (${\overset{⟶}{c}}_{j}$).

**Figure 3:**
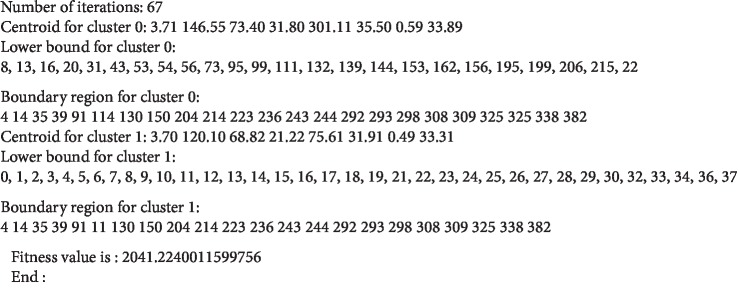
Snapshot of output RKM algorithm.

The rough k-means algorithm has stability and reliability for handling ambiguity. The rough k-means algorithm has clustered objects into lower bound and upper bound. The objects in the upper bound are ambiguous objects, whereas the objects in the lower bound are correct objects. The upper bound should not be empty, and the objects in the upper bound can belong to one or more upper bounds in the cluster numbers.  Figure 3 shows a snapshot of output obtained from the RKM algorithm to determine the ambiguous objects for improving the performance of machine learning algorithms. The objects in lower bound are correction objects, whereas the objects on boundary bound are ambiguous objects.

### 3.3. Classification Algorithms

In this section, conventional machine learning algorithms are discussed. The automatic classification, namely, naive Bayes (NB), support vector machine (SVM), K-nearest neighbor (KNN), and random forest tree, are presented to predict chronic diseases for enhancing healthcare systems.

#### 3.3.1. Support Vector Machine Algorithm

The support vector machine is used to analyze data as classification and regression. In the SVM algorithm, the data point is considered as *n*-dimensional space where there are a number of features of data, and the values of features are the values of a specific coordinate. The classification of data is achieved by finding the best difference between the classes of data using hyperplane. A support vector machine algorithm classifies data by separating the hyperactive plane of label training data. The SVM obtains lower error when the margin is large. In the present research work, two classes of chronic diseases are used. All types of kernel functions are applied to classify the chronic disease datasets in which radial basis function (RBF), along with kernel function, obtain high accuracy. The kernel function is applied and observed that the RBF function and kernel function are appropriate with the RKM algorithm to obtain good accuracy.

(1) $$\begin{array}{c} k\left(x,\bar{x}\right)=\exp\left(-\;\frac{\left\|x-\bar{x}\right\|}{2\sigma^{2}}\right), \end{array}$$

where ${\left\|x-\bar{x}\right\|}^{2}$ is the square Euclidean distance between two feature vectors and *σ* is a parameter.

#### 3.3.2. Naïve Bayes Algorithm

Naive Bayes algorithm is defined as a probabilistic method used to classify the dataset based on the well-known Bayes theorem of probability. The naïve Bayes classification algorithm works as prior probability, posterior probability, likelihood probability, and evidence probability. It normally uses probability distributions. The working Bayesian algorithm is as follows: Assume *A* = *A*_1_, *A*_2_, *A*_3_,…, *A*_*n*_ is regarded as the feature vector of chronic disease features, and the values of the features are *A*_1_, *A*_2_, *A*_3_,…, *A*_*n*_ and are considered as a number of features in the dataset. *C* indicates a class of chronic data as normal and abnormal. The Bayes equation is shown as follows:

  Conditional probability:

(2) $$\begin{array}{c} P\left(\left.C\right|A\right)=\frac{P\left(A,C\right)}{P\left(A\right)}, \end{array}$$

(3) $$\begin{array}{c} P\left(\left.C\right|A\right)=\frac{P\left(A,C\right)}{P\left(c\right)}. \end{array}$$

It is assumed that the predictor *A* on the given class *c* is independent of the values of other predictors, and it is known as conditional class independence. *P*(*C*|*A*) is the posterior probability of class *c*, given predictor (feature).

  Theorem is as follows:

(4) $$\begin{array}{c} P\left(\left.C\right|A\right)=\frac{P\left(C\right)\;.\;P\;\left(\left.A\right|C\right)}{P\left(A\right)}. \end{array}$$

#### 3.3.3. K-Nearest Neighbors Algorithm

A K-nearest neighbors algorithm is a simple machine learning algorithm, which uses the entire dataset in its training phase. KNN algorithm has low complexity in programming and implementation. The basic idea can be presented in a sample space when its nearest neighboring features belong to a category, and then the features belong to the same category. The KNN classification algorithm can be used with either a single or a multidimensional feature dataset and can find the closest features. It employs the Euclidean distance method for finding the closest point among the features.

(5) $$\begin{array}{c} D=\sqrt{\left(x_{1}-x_{2}\right)+{\left(y_{1}-y_{2}\right)}^{2}}. \end{array}$$

#### 3.3.4. Random Forest Algorithm

A decision tree algorithm is one of the powerful decisions. It is used to build the block of a random forest. It works to select the best split of an object from the dataset in each step. To reduce the high harnessed of variance, we can create multiple trees with various samples of datasets and combine this operation with bootstrap aggregation or bagging. The disadvantage of the bootstrap aggregation method is used to spill the values of each tree, which creates a problem in decision-making. Furthermore, it makes predictions of training data similar and mitigates the variance originally sought. Thus, the random forest algorithm can be further used for the classification and regression problems and for the overfitting of data as well. The selected attributes are measured by employing the information gain method to discover the value or the information from the entire dataset. The information gain method is calculated for each splitting attribute with selecting high gain attributes. It is assumed that *D* is the dataset.

(6) $$\begin{array}{c} \text{info}\left(D\right)=-\sum_{i=1}^{m}\left(p_{i}\log\;p_{i}\right), \end{array}$$

where *D* is the dataset, *i* = 1, 2,…, *m* is the class of dataset *D*, and the probability is *p*_*i*_

Let *B* be an attribute in dataset *D* and {*b*_1_, *b*_2_, *b*_3_,…, *b*_*n*_} are values of the attributes in *B*. Attributes are a partition for generating the amount of information from attributes.

(7) $$\begin{array}{c} \text{info}\left(D\right)\sum_{j=1}^{n}\left(\frac{\left|D_{j}\right|}{D}\right)*\text{info}\left(D_{j}\right). \end{array}$$

The attributes show the highest information as follows:

(8) $$\begin{array}{c} \text{Gain}\left(B\right)=\text{info}\left(D\right)-{\text{info}}_{B}\left(D\right). \end{array}$$

### 3.4. Performance Measurement

The performance measures are used to test and evaluate the proposed system. The accuracy, specificity and sensitivity, precision, recall, and *F*-score evaluation matrices have been employed to test the proposed model. The evaluation matrices are computed by using the equations (9)–(13) as described below. where we have true positive (TP), true negative (TN), false positive (FP), and false negative (FN).

#### 3.4.1. Accuracy

Accuracy is the number of correct predictions made by the model over all kinds of predictions made. It is calculated as the total number of correct labels (TP + TN) divided by the total number of chronic disease datasets (*P* + *N*):

(9) $$\begin{array}{c} \text{accuracy}=\frac{\text{TP}+\text{TN}}{\text{TP}+\text{FP}+\text{FN}+\text{TN}}. \end{array}$$

#### 3.4.2. Specificity

Specificity (also called the true negative rate) is a measure that tells us about the percentage of patients who do not suffer from chronic diseases, which are predicted by the model as not chronic diseases:

(10) $$\begin{array}{c} \text{specificity}=\frac{\text{TN}}{\text{TN}+\text{FP}}\times 100\%. \end{array}$$

#### 3.4.3. Sensitivity

Sensitivity (also called the true positive rate, the recall, or probability of detection) is the measure that tells about the percentage of patients who actually suffer from chronic diseases, which are diagnosed by the classification algorithms on chronic diseases:

(11) $$\begin{array}{c} \text{specificity}=\frac{\text{TP}}{\text{TP}+\text{FN}}\times 100\%. \end{array}$$

#### 3.4.4. Precision

Precision is a measure that tells about the proportion of patients that we diagnosed as having chronic diseases, actually had chronic diseases. It is known as positive predictive value (PPV):

(12) $$\begin{array}{c} \text{precision}=\frac{\text{TP}}{\text{TP}+\text{FP}}\times 100\%. \end{array}$$

#### 3.4.5. *F*1-Score

*F*1-score (also called the precision, *F*-score, *F*-measure, and recall) is the harmonic mean (average) of the precision and recall:

(13) $$\begin{array}{c} F1\;-\text{score}=\frac{2\;*\text{precision}*\text{sensitivity}}{\text{precision}+\text{sensitivity}}\%100. \end{array}$$

## 4. Experimental Results and Discussion

Therefore, the rough K-means algorithm is applied for improving the classification of chronic diseases. It is used to determine the ambiguous objects that have obstructed the classification algorithms. It has further experimented with various standard chronic datasets. It is aimed here to improve the diagnosis of chronic diseases. In the beginning, the conventional classification algorithms are applied to predict chronic diseases. However, it is observed that the obtained results were not appropriate. From the obtained results, it is noted that there are ambiguous objects that decrease the accuracy of machine learning algorithms. One of the biggest challenges that we have faced within the implementation of the proposed system is the ambiguity embedded in the variable of the standard dataset. For this reason, the RKM algorithm is considered to handle these ambiguous objects so that the accuracy of the classification algorithms can be improved. The RKM algorithm is appropriately designed for detecting the ambiguity in the chronic disease datasets. The experimental results have shown that the performance of the proposed system is better than that of the conventional models. For measuring and evaluating the performance of the proposed system, the performance measures are applied. The standard evaluation matrices, namely, accuracy, specificity, sensitivity, precision, and *F*-score have been presented to test the proposed system against the existing machine learning techniques. Moreover, for validating the proposed system, the datasets are divided into 70% train and 30% test. Numerous experiments have attempted to evaluate the proposed system. The results of machine learning algorithms and enhanced model to the various datasets are presented as follows:

### 4.1. Classification Results of Diabetics Disease

In this section, different experiments of classification algorithms with the enhanced proposed system have been conducted. The soft computing rough K-means algorithm is used to handle ambiguous objects. The ambiguous objects in chronic disease datasets have reduced the performance of machine learning algorithms. When applying the classification algorithm on the original diabetics data, it is observed that the results are not favourable. From the data, it is investigated that there are ambiguous objects that hinder the classification algorithms. The diabetes data contain seven instances and two classes. These ambiguous objects are examined by RKM clustering to assist in determining the exact class of ambiguous diseases or the closest one. The dataset has been clustered for two clusters corresponding into two classes that are labelled variables in datasets. The RKM algorithm has clustered the ambiguous objects into upper approximation and lower approximation. Those objects that belong to upper approximation, which belongs to one or more cluster numbers, are excluded. Among 768 instances, 718 instances are clustered as a lower approximation. Moreover, the remaining objects are clustered as an upper approximation and are considered as ambiguous objects as well. The ambiguous objects have been denied from the data. The classification algorithm is applied to process the data in a lower approximation for diagnosing the diseases.  Table 4 shows the results obtained from the RKM algorithm used for discovering the ambiguous objects.

**Figure 4:**
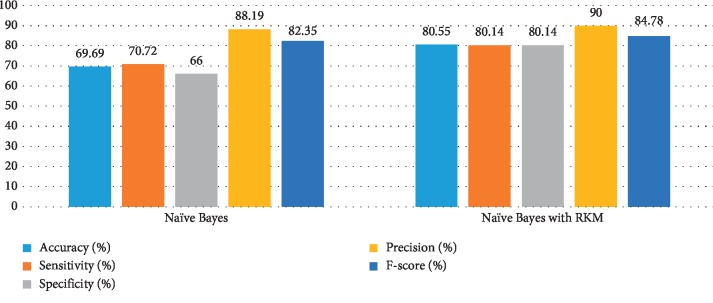
Comparison results of existing naïve Bayes classifier and naïve Bayes using RKM algorithm for diabetic diseases.

**Figure 5:**
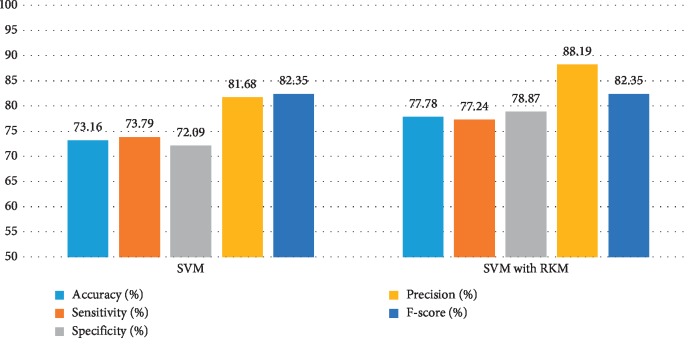
Comparison results of existing SVM classifier and SVM using RKM algorithm for diabetic diseases.

**Figure 6:**
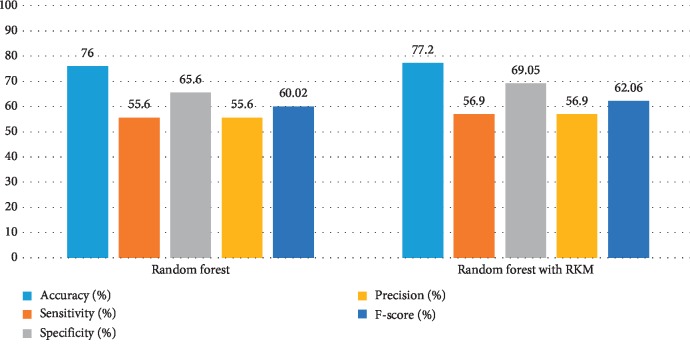
Comparison results of existing random forest classifier and random forest using RKM algorithm for diabetic diseases.

**Figure 7:**
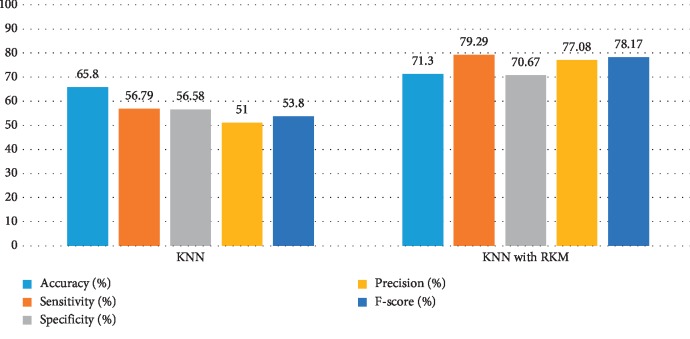
Comparison results of existing KNN classifier and KNN using RKM algorithm for diabetic diseases.

Table 5 shows the results of the classification algorithm, namely, naïve Bayes, SVM, random forest tree, and KNN. It is observed that the obtained results are needed to improve. The rough K-means is applied to enhance the existing machine learning.  Table 6 shows the results of machine learning techniques with RKM algorithm. The rough K-means is used to deal with ambiguous objects. It is observed that the RKM algorithm has improved the results of the classification algorithms. The results of naïve Bayes with RKM are 80.55%, 80.14%, 80.14%, 90%, and 84.78% in terms of accuracy, sensitivity, specificity, precision, and F-score, respectively. Similarly, the results of SVM with RKM approaches are 77.78, 77.24, 78.87, 88.19, and 82.35 corresponding to accuracy, sensitivity and specificity, precision, and *F*-score, in that order. The results of random forest with RKM are 77.20, 56.09, 69.05, and 62.0. Furthermore, the obtained results by using KNN with RKM are 71.30%, 79.29%, 56.58%, 77.08%, 77.08%, and 78.70%. Finally, from the obtained data, it is investigated that the classification algorithm is improved by using the RKM algorithm. Figures 4–7 show the performance of the classification algorithms with the RKM algorithm.

### 4.2. Classification Results of Kidney Disease

This section demonstrates the classification of kidney diseases with the help of machine learning algorithms and the enhanced proposed system.  Table 7 shows the results obtained from the RKM algorithm to figure out the ambiguous objects. Kidney diseases contain 400 instances. The data are clustered into two clusters. The rough K-means clusters data into upper approximation and lower approximation. The objects that have been clustered in lower approximation are 174 instances. Those objects that belong to lower approximation are regarded as approved objects because they belong to the same cluster numbers. The remaining objects, that is, 226 objects, are clustered in upper approximation, which is considered as ambiguous objects.

**Figure 8:**
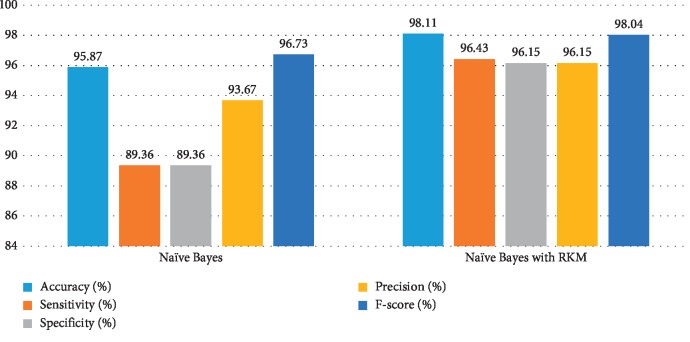
Comparison of results of existing naïve Bayes classifier and naïve Bayes using RKM algorithm for kidney diseases.

**Figure 9:**
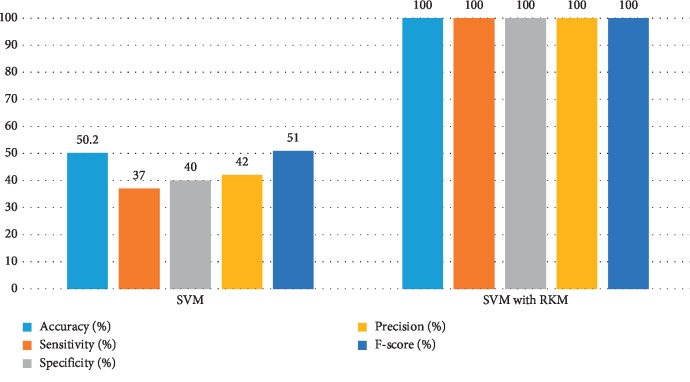
Comparison of results of existing SVM classifier and SVM using the RKM algorithm for kidney diseases.

**Figure 10:**
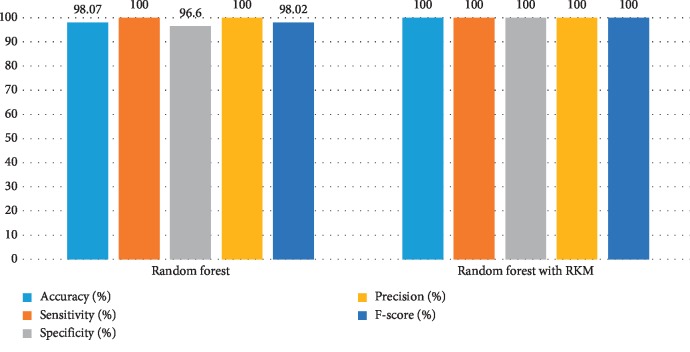
Comparison of results of existing random forest classifier and random forest using the RKM algorithm for kidney diseases.

**Figure 11:**
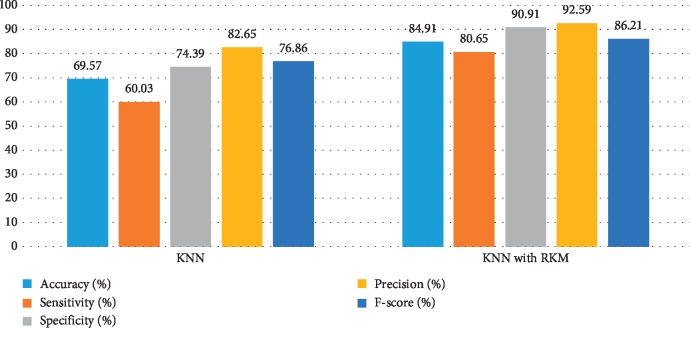
Comparison results of existing KNN classifier and KNN using the RKM algorithm for kidney diseases.

Table 8 shows the performance of existing machine learning algorithms, namely, naïve Bayes, SVM, random forest tree, and KNN. It is observed that there is a possibility for improving the classification algorithms if they handle ambiguous objects. It is also noted that the RKM algorithm has improved the results of the classification algorithms.  Table 9 shows results of machine learning techniques with the RKM algorithm. The results of naïve Bayes with RKM are 98.11%, 96.43%, 96.15%, 96.15%, and 98.04.78%, with respect to the evaluation matrices. Similarly, results of SVM with RKM approaches are 100%, 100%, 100, 100%, and 100% in terms of accuracy, sensitivity and specificity, precision, and F-score, respectively. The results of random forest with RKM are 100%, 100%, 100, 100%, and 98.02%. Furthermore, the obtained results by using KNN with RKM are 84.91%, 80.65%, 90.91%, 92.59%, and 86.21%. Lastly, from the obtained results, it is found that the classification algorithm is improved by using the RKM algorithm. Figures 8–11 display the performance of the classification algorithms with the RKM algorithm for predicting kidney diseases.

### 4.3. Classification Results of Cancer Disease

This section shows the classification of cancer disease using the existing machine learning algorithms and the proposed system using the RKM algorithm. The cancer data contain 569 instances that are classified into two classes such as benign and malignant. The soft computing RKM clustering algorithm is used to handle ambiguous objects. It clusters data into two clusters according to the class label of data. The RKM technique has clustered data into lower approximation and upper approximation. The objects belonged to the lower approximation are appropriate objects, and are processed by using a machine learning algorithm. However, the objects that have been clustered into upper approximation are considered as ambiguous ones. The RKM is clustered into 539 objects in the lower approximation, and the remaining objects are in the upper approximation.  Table 10 demonstrates the results of the RKM algorithm. Subsequently, machine learning is applied to diagnose cancer as benign and malignant.

**Figure 12:**
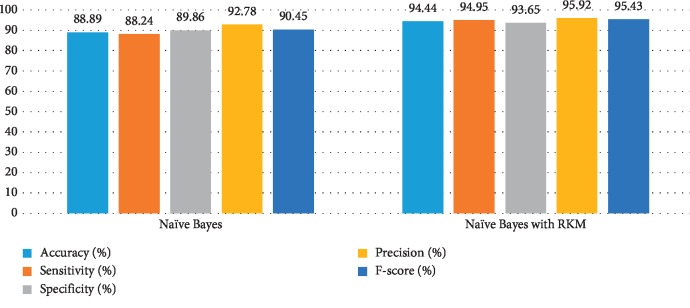
Comparison of results of the existing naïve Bayes classifier and naïve Bayes using the RKM algorithm for cancer disease.

**Figure 13:**
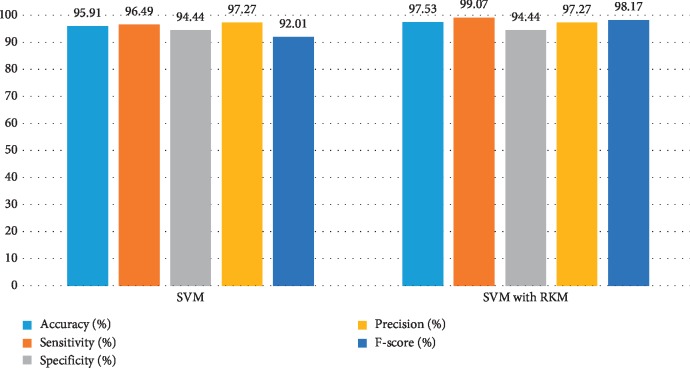
Comparison of results of the existing SVM classifier and SVM using the RKM algorithm for cancer disease.

**Figure 14:**
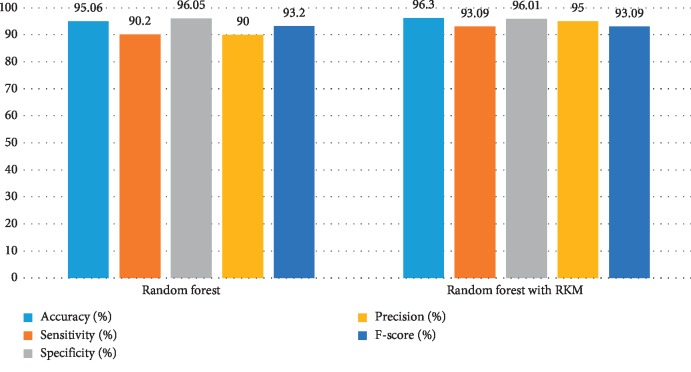
Comparison of results of the existing random forest classifier and random forest using the RKM algorithm for cancer disease.

**Figure 15:**
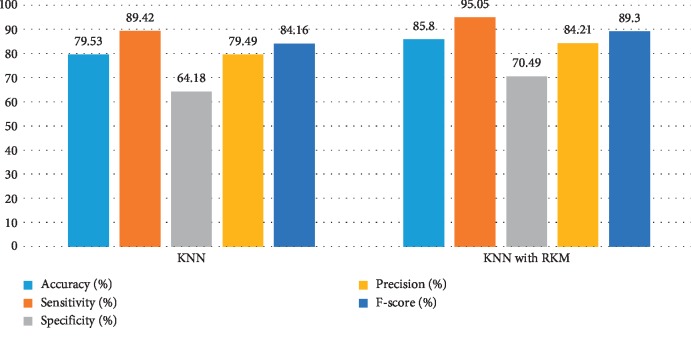
Comparison of results of the existing KNN classifier and KNN using the RKM algorithm for cancer disease.

Table 11 shows the obtained results of conventional machine learning for the classification of cancer disease. It is noted that the results need more improvement, and the RKM algorithm is applied to enhance the existing machine learning algorithm.  Table 12 shows results analysis of the proposed model. The results of naïve Bayes with RKM are 94.44%, 94.95%, 93.65%, 95.92%, and 95.43% in terms of accuracy, sensitivity, specificity, precision, and F-score. Similarly, the results of SVM with RKM approaches are 97.53%, 99.07%, 94.44%, 97.27, and 98.17% regarding accuracy, sensitivity, specificity, precision, and F-score, respectively. The results of random forest with RKM are 96.30%, 93.09%, 96.01%, 95%, and 93.09%. Furthermore, the obtained results by using KNN with RKM are 85.80%, 95.05%, 70.49%, 84.21%, and 89.30%. Finally, from the obtained results, it is investigated that the classification algorithms are improved by using a soft clustering RKM algorithm. Figures 12–15 display the performance of the classification algorithms with the RKM algorithm for the diagnosis of cancer disease. From graphic representations that are shown, it can be noted that the proposed system is better.

## 5. Comparative Analysis

In this section, a comparative analysis between the proposed model and some of the other state-of-the-art work is used in the same datasets. The comparison is very important because it examines the results of the proposed model. The accuracy metric is used to compare the proposed model with the existing classification algorithms.  Table 13 shows the results of the proposed system and the existing neural network approach. It is investigated that the results of the proposed system are better than those of the existing neural network approach.

## 6. Conclusion

The performance of the existing machine learning is thwarted from diagnosing chronic diseases because of the availability of ambiguous objects. These ambiguous objects show traits in more than one class. To identify and process the ambiguous objects explicitly, we have demonstrated the noncrisp RKM clustering that can handle these ambiguous objects to improve the accuracy of classification algorithms. The framework of the proposed system lies in its use of a soft clustering algorithm, namely, rough K-means that can be employed for modelling ambiguity. The rough K-means clustering can assist in determining the exact class of the ambiguous objects or the approximate ones. It is observed that the RKM algorithm has increased the performance of the conventional machine algorithms to predict chronic diseases. The ambiguous objects are excluded from chronic dataset. Therefore, the RKM algorithm clustered the data into lower and upper approximation. The objects clustered in lower approximation are considered as appropriate objects. Additionally, the objects that belong to the upper approximation are denied and considered as ambiguous objects. The objects that belong to the lower approximation are proposed by using machine learning algorithms to predict chronic diseases. The experimental results demonstrate that the proposed system is successfully employed for the diagnosis of chronic diseases. Comparative analysis results between existing machine learning algorithms and the proposed system are presented. Moreover, it is observed that the results of the proposed system are superior in terms of accuracy, specificity, sensitivity, precision, recall, and F-score performance measures. Identifying common web search activity behaviour is regarded as a proxy for chronic disease risk factors using machine learning algorithms can be considered in future work.

## Figures and Tables

**Table 1:** Features of diabetic dataset.

Feature name	Categories
Pregnancies	Numeric
Glucose	Numeric
Blood pressure	Numeric
Skin thickness	Numeric
Insulin	Numeric
BMI	Numeric
Diabetes pedigree function	Numeric
Age	Numeric
Class	Nominal, diabetics, not diabetics (0, 1)

**Table 2:** Features of cancer dataset.

Feature name	Categories
Id	Numeric
Radius mean	Numeric
Texture_mean	Numeric
Perimeter_mean	Numeric
Area_mean	Numeric
Smoothness_mean	Numeric
Compactness_mean	Numeric
Concavity_mean	Numeric
CONCAVE points_mean	Numeric
Symmetry_mean	Numeric
Fractal_dimension_mean	Numeric
Radius_se	Numeric
Texture_se	Numeric
Perimeter_se	Numeric
Area_se	Numeric
Smoothness_se	Numeric
Compactness_se	Numeric
Concavity_se	Numeric
Concave points_se	Numeric
Symmetry_se	Numeric
Fractal_dimension_se	Numeric
Radius_worst	Numeric
Texture_worst	Numeric
Perimeter_worst	Numeric
Area_worst	Numeric
Smoothness_worst	Numeric
Compactness_worst	Numeric
Concavity_worst	Numeric
Concave points_worst	Numeric
Symmetry_worst	Numeric
Fractal_dimension_worst	Numeric
Diagnosis	Nominal (*M* = malignant and *B* = benign)

**Table 3:** Features of kidney dataset.

Feature name	Categories
Age	Numeric
bp: blood pressure	Numeric
sg: specific gravity	Nominal: 1.005, 1.010, 1.015, 1.020, 1.025
al: albumin	Nominal: 0, 1, 2, 3, 4, 5
su: sugar	Nominal: 0, 1, 2, 3, 4, 5
rbc: red blood cells	Nominal: 0, 1
pc: pus cell	Nominal: 0, 1
pcc: pus cell clumps	Nominal: 0, 1
ba: bacteria	Nominal: 0, 1
bgr: blood glucose random	Numeric
bu: blood urea	Numeric
sc: serum creatinine	Numeric
sod: sodium	Numeric
pot: potassium	Numeric
hemo: hemoglobin	Numeric
pcv: packed cell volume	Numeric
wc: white blood cell count	Numeric
rc: red blood cell count	Numeric
htn: hypertension	Nominal
dm: diabetes mellitus	Nominal
cad: coronary artery disease	Nominal
appet: appetite	Nominal
pe: pedal edema	Nominal
ane: anemia	Nominal
class: class	Nominal: CKD, not CKD

**Table 4:** Results of RKM algorithm for handling ambiguous objects in the diabetic dataset.

Cluster number	Lower approximation	Upper approximation
Cluster 1 Cluster 2	718	50

**Table 5:** Results of existing machine learning for diagnosing diabetic diseases.

Model	Accuracy (%)	Sensitivity (%)	Specificity (%)	Precision (%)	*F*-score (%)
Naïve Bayes	69.69	70.72	66	88.19	82.35
SVM	73.16	73.79	72.09	81.68	82.35
Random forest	76	55.6	65.6	55.6	60.02
KNN	65.80	56.79	56.58	51	53.80

**Table 6:** Results of machine learning after handling ambiguous objects for diagnosing diabetic diseases.

Model	Accuracy (%)	Sensitivity (%)	Specificity (%)	Precision (%)	*F*-score (%)
Naïve Bayes with RKM	80.55	80.14	80.14	90	84.78
SVM with RKM	77.78	77.24	78.87	88.19	82.35
Random forest with RKM	77.20	56.9	69.05	56.9	62.06
KNN with RKM	71.30	79.29	70.67	77.08	78.17

**Table 7:** Results of RKM algorithm for handling ambiguous objects in kidney dataset.

Cluster number	Lower approximation	Upper approximation
Cluster 1 Cluster 2	174	226

**Table 8:** Results of existing machine learning for diagnosing kidney diseases.

Model	Accuracy (%)	Sensitivity (%)	Specificity (%)	Precision (%)	*F*-score (%)
Naïve Bayes	95.87	89.36	89.36	93.67	96.73
SVM	50.2	37.0	40.0	42.0	51
Random forest	98.07	100	96.6	100	98.02
KNN	69.57	60.03	74.39	82.65	76.86

**Table 9:** Results of machine learning after handling ambiguous objects for diagnosing kidney diseases.

Model	Accuracy (%)	Sensitivity (%)	Specificity (%)	Precision (%)	*F*-score (%)
Naïve Bayes with RKM	98.11	96.43	96.15	96.15	98.04
SVM with RKM	100	100	100	100	100
Random forest with RKM	100	100	100	100	100
KNN with RKM	84.91	80.65	90.91	92.59	86.21

**Table 10:** Results analysis of machine learning algorithm using the RKM algorithm for the diagnosis of cancer disease.

Cluster number	Lower approximation	Upper approximation
Cluster 1 Cluster 2	174	226

**Table 11:** Results of existing machine learning for the diagnosis of cancer disease dataset.

Model	Accuracy (%)	Sensitivity (%)	Specificity (%)	Precision (%)	*F*-score (%)
Naïve Bayes	88.89	88.24	89.86	92.78	90.45
SVM	95.91	96.49	94.44	97.27	92.01
Random forest	95.06	90.2	96.05	90	93.2
KNN	79.53	89.42	64.18	79.49	84.16

**Table 12:** Results of machine learning after handling ambiguous objects for the diagnosis of cancer disease dataset.

Model	Accuracy (%)	Sensitivity (%)	Specificity (%)	Precision (%)	*F*-score (%)
Naïve Bayes with RKM	94.44	94.95	93.65	95.92	95.43
SVM with RKM	97.53	99.07	94.44	97.27	98.17
Random forest with RKM	96.30	93.09	96.01	95.0	93.09
KNN with RKM	85.80	95.05	70.49	84.21	89.30

**Table 13:** Results of the proposed system against the existing neural network approach.

Technique	References	Accuracy (%)	Diseases
ANN	[45]	80.4	Kidney
General regression neural network	[46]	80.20	Diabetes
Back propagation neural network	[47]	95.03	Kidney
BPNNs	[48]	92.84	Breast cancer
*Proposed model*		*100*	*Kidney*
*Proposed model*		*80.55*	*Diabetes*
*Propose model*		*97.53*	*Breast cancer*

## Data Availability

The data used to support the findings of this study are available from the corresponding author upon request.

## Supplementary Materials

Supplementary Materials The chronic disease datasets have been collected from different resources as follows: 1. Diabetics disease dataset: the diabetes data collected from the machine learning repository contained nine attributes, eight features, and one class. This dataset has been gathered from an automatic electronic recording device and paper records. 2. Breast cancer disease dataset: the cancer data collected from the Kaggle contained nine attributes, 30 features, and 1 class. These features are obtained from digitized images of breast cancer [38]. 3. Kidney disease dataset: the collected kidney data from the Kaggle contained 26 attributes, 24 features, and 1 class [38].
